# Annotations

**Published:** 1894-02-24

**Authors:** 


### ANNOTATIONS.

### Henry VIII. to the Rescue.

THE lapse of time as well as "adversity" may "make us acquainted with strange bed-fellows." Mr. H. Newton, a Keighley herbalist, has been fined twice £20, after the consideration of his case by his Honour Judge Gates, Q.C., and a jury, for practising as a medical man. Mr. Newton, it appears, is a member of the Keighley Town Council, so high does unqualified practice fly in the manufacturing towns of West Yorkshire. The amusing part of this case is, that notwithstanding the loose and wobbling condition of the law with regard to unqualified medical practice, the defendant's solicitor could find no solid foundation on which to rest a defence without raking up an old and practically obsolete statute of Henry VIII., bearing date 1542. The husband of Catherine of Arragon was a person who had a fine appreciation of the comical, and he would certainly have appended his royal signature to the statute with additional gusto if he could have foreseen that more than three hundred years after his death it would be fished up by a country solicitor for the defence of a Keighley herbalist. That is the funny side. The serious side is that there was no solid ground for the prosecution to stand upon either, except the Apothecaries Act of 1815. All this is very humiliating to the medical profession. We claim, and with justice, to be the most scientific, and, from a modern point of view, the most cultured of all the professions. To a large extent we lead the science of the times. And yet any policeman, or blacksmith, or herbalist may assume the rights and dignities of our calling, and there is not a single statute which will enable us to say him nay if he be a discreet person. The Medical Defence Union is to be congratulated upon ts success in this action; but when will it and other similar organisations obtain for the medical profession such an alteration in the law of the land as will at once conserve our own self-respect and the best health interests of the community?

### Our Failing Teeth.

THE dentists of the period, who are nothing if not scientific, raise a note of alarm about the growing tendency to decay of the teeth of the present and the coming generations. Dental caries is said to be increasing in an "extraordinary and alarming" manner. Each succeeding generation shows a poorer and poorer quality of teeth. This the writer can confirm to some extent by the experience of four generations of his own family. At one extreme was a grandfather of eighty-six, who died less than a score of years ago, with a mouth full of absolutely perfect teeth; at the other is the great-granddaughter of that old gentleman who, at ten years of age, requires six of her teeth "filling" at the present moment. What can be the cause of this very unpleasant and even alarming condition of things? The dentists tell us that "dental caries marches hand in hand with civilisation." If that be so, we can only devoutly wish that civilisation would find a more encouraging and comfortable companion. But why does civilisation insist upon destroying our teeth? Because, say the dentists, "The increasing perfection of the culinary art, by reducing the work of the masticating organs to a minimum," causes both teeth and jaws to atrophy and decay. So then it is the cook, the scientific cook of the schools of cookery who, in the last resort, is at fault. Even our domesticated animals, our cats and dogs, are losing the excellence of their teeth for the same reason, and we shall no doubt soon have dentists among the veterinary surgeons, as well as among the more august professors of the art of human medicine. These be uncomfortable prophesyings! Can anything be done? A little, say the dentists. We must all go in for brown bread. Wholemeal bread alone contains in quantity the fluorine which is so necessary for the hardness and permanence of the teeth. Wholemeal bread it must be, then, at morning, at noon, and at night, if we would avoid the pangs of toothache, and the pains of dentistry, and save our precious teeth.

### Evening Out-patients.

FAIRPLAY is one of the finest rules of the game of life, as it is of the game of cricket. The general practitioner has long experienced a minimum of fairplay at the hands of the hospitals, but it is now proposed to bandage his eyes and to make him bat with one hand, and that the left hand. It is proposed to open the out-patient departments of hospitals in the evening, and to make no additional regulations for keeping out those who are able to pay. This is a question we have earnestly considered for a long time. A most plausible face has been put upon it by those who love to get the best doctoring and to pay nothing for it—namely, by the well-paid workmen, who take all that the medical profession has to give them and then wonder that there are so many weak-minded persons in the profession who do not understand even the elements of political economy. The workman accepts the hospital doctor's treatment, but thinks him a fool for being so ready to give it for nothing. Now if the working class were so abjectly poor and helpless that they could neither pay their doctors nor afford to lose an hour's wages by going to the hospital in the daytime, we should be the very first, political economy notwithstanding, to ask for the opening of out-patient departments in the evening. But we know that it is not so. Both workingmen and workingwomen can go to the hospital in the daytime if they wish. Our mature conviction is that to open the out-patient departments in the evening will be to strike another grievous blow at the general practitioner, and to do the working classes themselves—especially the unmarried among them—more harm than good. There is a class of hospital doctors who are prepared to sacrifice their professional brethren on the altar of their own ambition without a moment's hesitation. These are not, we are happy to think, the better class of consultants, but the struggling nobodies who are anxious to be somebodies. But is the whole working profession to be sacrificed to the ignorant and unworthy ambition of this vain and foolish handful? The thoughtful, and the just among hospital physicians and surgeons should take this question to heart, and should stand by the profession as a whole, even at the risk of some temporary unpopularity. But at any rate the general practitioners in the poorer neighbourhoods must promptly combine, and by union and loyalty to one another put a check upon these fatal hospital encroachments on their life market.

